# Formation of Paramagnetic Defects in the Synthesis of Silicon Carbide

**DOI:** 10.3390/mi14081517

**Published:** 2023-07-28

**Authors:** Nain Mukesh, Bence G. Márkus, Nikoletta Jegenyes, Gábor Bortel, Sarah M. Bezerra, Ferenc Simon, David Beke, Adam Gali

**Affiliations:** 1 Institute of Physics, ELTE Eötvös Loránd University, Egyetem tér 1-3., H-1053 Budapest, Hungary; 2 Wigner Research Centre for Physics, Institute for Solid State Physics and Optics, H-1525 Budapest, Hungary; 3 Stavropoulos Center for Complex Quantum Matter, Department of Physics and Astronomy, University of Notre Dame, Notre Dame, IN 46556, USA; 4 Department of Physics, Institute of Physics and ELKH-BME Condensed Matter Research Group, Budapest University of Technology and Economics, Műegyetem Rakpart 3., H-1111 Budapest, Hungary; 5 Department of Physical Chemistry and Materials Science, Faculty of Chemical Technology and Biotechnology, Budapest University of Technology and Economics, Műegyetem Rakpart 3., H-1111 Budapest, Hungary; 6 Department of Atomic Physics, Institute of Physics, Budapest University of Technology and Economics, Műegyetem Rakpart 3., H-1111 Budapest, Hungary

**Keywords:** solid state quantum defects, electron paramagnetic resonance (EPR), photoluminescence

## Abstract

Silicon carbide (SiC) is a very promising platform for quantum information processing, as it can host room temperature solid state defect quantum bits. These room temperature quantum bits are realized by paramagnetic silicon vacancy and divacancy defects in SiC that are typically introduced by irradiation techniques. However, irradiation techniques often introduce unwanted defects near the target quantum bit defects that can be detrimental for the operation of quantum bits. Here, we demonstrate that by adding aluminum precursor to the silicon and carbon sources, quantum bit defects are created in the synthesis of SiC without any post treatments. We optimized the synthesis parameters to maximize the paramagnetic defect concentrations—including already established defect quantum bits—monitored by electron spin resonance spectroscopy.

## 1. Introduction

Quantum technology is now a driving force in the fields of materials science and nanotechnology. Silicon carbide (SiC) is one of the most promising material platforms for the integration of defect quantum bits which are the elementary building blocks of quantum information processing. Most of the promising defects are vacancy-related, created by irradiation techniques [1,2] or laser writing [3]. Isolated silicon vacancy in its negative charge state (V_Si_^−^) is a well-known fundamental defect in SiC associated with single photon emitters with coherently controllable electron spin at room temperature in the hexagonal polytypes [4,5,6,7,8,9,10,11,12]. Divacancy (V_C_-V_Si_) is a defect complex of adjacent silicon and carbon vacancies [13,14] created by annealing of irradiated SiC. Its neutral charge state is a color center [15,16,17] with a triplet ground state for which coherent manipulation was firstly demonstrated among the quantum defects in SiC [18,19]. In hexagonal polytypes, V_Si_^−^ emits in the region of 860–950 nm [5] whereas the neutral divacancy has a longer wavelength emission at around 1100–1300 nm [20]. V_Si_ and V_C_-V_Si_ emitters may be applied as fluorescence probe sensors too [21,22].

Beside the established defect quantum bits, bright single photon emitters were reported in bulk hexagonal SiC [23]. A bright single photon emitter was found in SiC nanoparticles (NPs) too at 600–700 nm called E-center [24]. The origin of the emitter was associated with the carbon antisite-vacancy complex (V_C_-C_Si_) that has a characteristic electron paramagnetic resonance (EPR) signal in the hexagonal and cubic polytypes of SiC [25,26]. We note that the V_Si_ and V_C_-C_Si_ defects may be transformed into each by annealing, as these two defect configurations can be described by moving one carbon atom from the lattice site in the V_Si_ defect into the vacant site with leaving the carbon-vacancy (V_C_) behind [23,27,28]. We note that no emission has been reported for V_C_ so far, but it has well-known EPR centers in its positive charge state in the hexagonal and cubic polytypes [26,29].

The irradiation and laser writing techniques have the advantage of being able to create the quantum bit vacancy defects at a desired location in the crystal. However, these techniques also produce many unwanted defects in addition to the target quantum bit defects. These defects produce uncontrolled strain and fluctuating charges under illumination around the defect quantum bits with creating undesired inhomogeneous broadening in the fluorescence spectrum and lifetime, and they can significantly reduce the spin coherence times [22]. We have recently developed a fully-chemical method in which the point defects are created during the synthesis of large SiC nanocrystals of an average size at about 100 nm [30] where the SiC crystal structure is dominated by the cubic polytype with dense hexagonal inclusions [31]. The proof of principle of this method was already demonstrated in our previous study [30]. In this technique, aluminum (Al) is applied as a precursor in the synthesis of SiC. During the reaction, Al basically removes Si atom from the SiC lattice. Therefore Si-vacancies can form, and it eventually dissolves completely without leaving any side product [32,33]. These defective large SiC nanocrystals were etched into ultrasmall (4 nanometers sized) SiC nanocrystals and annealed which transformed the Si-vacancy related defects to divacancy defects inside the ultrasmall SiC nanocrystals [30]. These results may contribute to the formation of nonperturbative bioagents for quantum sensing and efficient hyperpolarization.

The defect spin concentration in the initially synthesized large SiC nanocrystals is essential in order to realize the quantum sensors at high yields. However, this issue has not yet been investigated in details as a function of the parameters of the applied synthesis technique. The problem with most of the methods in terms of the defect concentration, when the size of the particles is especially at the nanoscale is that the defect types are dependent on reaction conditions. In this study, we report a thorough study about monitoring the defect spins concentrations in the SiC crystals as a function of the key synthesis parameters in order to maximize the concentration of the defect quantum bits. In particular, we monitored the target defect concentration by varying the Al composition and synthesis reaction time.

## 2. Experimental

### Sample Preparation

Here we briefly describe the process. Silicon (Si), black carbon, and polytetrafluoroethylene (PTFE) powders are milled by ball mill, and Al powder is also added to the precursor [32,33,34]. The mixture is then heated during chemical combustion synthesis [35] for the reaction of Si and C to form SiC. In this study, 1:1 atomic ratio of Si and C was used and 5 wt% PTFE was added as a promoter. Precursor mixing was carried out in a pulverisette7 (Fritsch, Idar-Oberstein, Germany) ball mill in an ethanol suspension (RPM 500, 8 min total milling time). The resulting slurry was dried at 80 °C. Approximately 2.5 g of dried powder was loaded in a graphite crucible. It was then placed in an induction furnace (Stanelco STX25-DF1). The synthesis time was varied as per the required feature observation of the material. The powder appears gray-black pigment contrast with grayer contour with increasing reaction time. To clean the SiC samples from the reactants, the powder was annealed at 650 °C for 10 h then the remaining powder (SiC + Si) was eventually cleaned with HF:HNO_3_:H_2_O mixture (15.2 mL of cc HF, 9.4 mL cc of HNO_3_, 100 mL H_2_O) per 5 g of powder in order to remove the extra silicon. Finally, the sample was washed with DI water and dried at 140 °C.

## 3. Analysis

**2.3(a)** The structural properties of SiC nanoparticles, e.g., crystallite size, crystal structure, SiC polytypes and presence of hexagonal inclusions were identified by powder X-ray diffraction (**XRD**) with Huber G670 Guinier Imaging Plate Camera (Rimsting, Germany). CuKα1 radiation was used to measure diffractograms. The hexagonal inclusions can be observed as non-cubic polytypes of SiC. The cubic polytype is labeled as 3C-SiC in the context.

**2.3(b) Photoluminescence Spectroscopy**. Photoluminescence (PL) was also measured by integrated sphere-based system which is an omnidirectional photons collector. PL spectra were collected with an Ocean Optics QE spectrometer. A 785 nm continuous diode laser (Roithner LaserTechnik GmbH, Vieanna, Austria) was used for excitation. Data were collected by OceanView software (https://www.oceaninsight.com, accessed on 24 July 2023).

**2.3(c)** We applied scanning electron microscope with energy dispersive spectroscopy (i.e., **SEM (EDS)**) for characterization of morphologies and elemental analysis (TESCAN MIRA3, Brno, Czech Republic) by using silicon substrate of thickness 2 mm. The SiC samples were drop casted with a thickness of ca 1 mm onto the Si substrate to avoid collection of X-ray photons originated from the substrate.

**2.3(d)** Paramagnetic spin species were studied using a benchtop **EPR** spectrometer (MiniScope MS-400, Berlin, Germany). EPR is a sensitive and versatile analytical tool to detect paramagnetic defects. We carried out cw EPR measurements, where the frequency is locked to the microwave cavity (rectangular TE_102_, $f_{res}\approx 9.38$ GHz), while the magnetic field is swept between 500 and 6000 G. The corresponding resonant field for g ≈2 is at ~0.335 T. We used a sufficiently low microwave power of 2.51 mW and a modulation of 300 mG to avoid saturation and overmodulation, respectively. For each measurement, approximately 5 mg of material was loaded into clean quartz tubes with an outer diameter of 5 mm (Wilmad^®^ 710-SQ-250, Vineland, NJ, USA). Data was analyzed using the EasySpin software (https://easyspin.org/, accessed on 24 July 2023) [36] to simulate different types of defects and to calculate their relative contributions.

## 4. Results and Discussion

**Figure 1:**
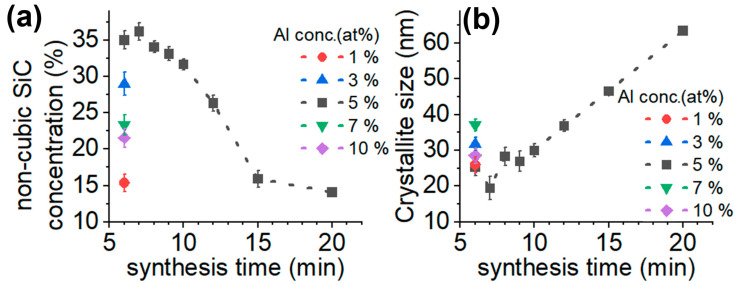
Crystalline structure of synthesized SiC. (**a**) Non-cubic SiC phase concentration and (**b**) crystallite size as a function reaction time for SiC powders prepared with various Al contents. The data are from Rietveld refinement of XRD results.

Figure 1 shows the crystallite size and polytype distribution as a function of reaction time and Al concentration in the precursor, calculated from XRD data by Rietveld full profile analysis. As can be seen, the crystallite size remains about 25 nm at the first 10 min of reaction time, and then it monotonously increases. The smallest crystallite sizes can be achieved at 5% Al concentration. The reaction of Si and C in an induction furnace creates 3C-SiC, dominantly, with some hexagonal phases. The concentration of the non-cubic SiC phase is proportional to the Al concentration in the samples while it is inversely proportional to the reaction time. We found an optimum at 5% Al concentration, where the concentration of the non-cubic phases reaches a maximum with the smallest particle size.

**Figure 2:**
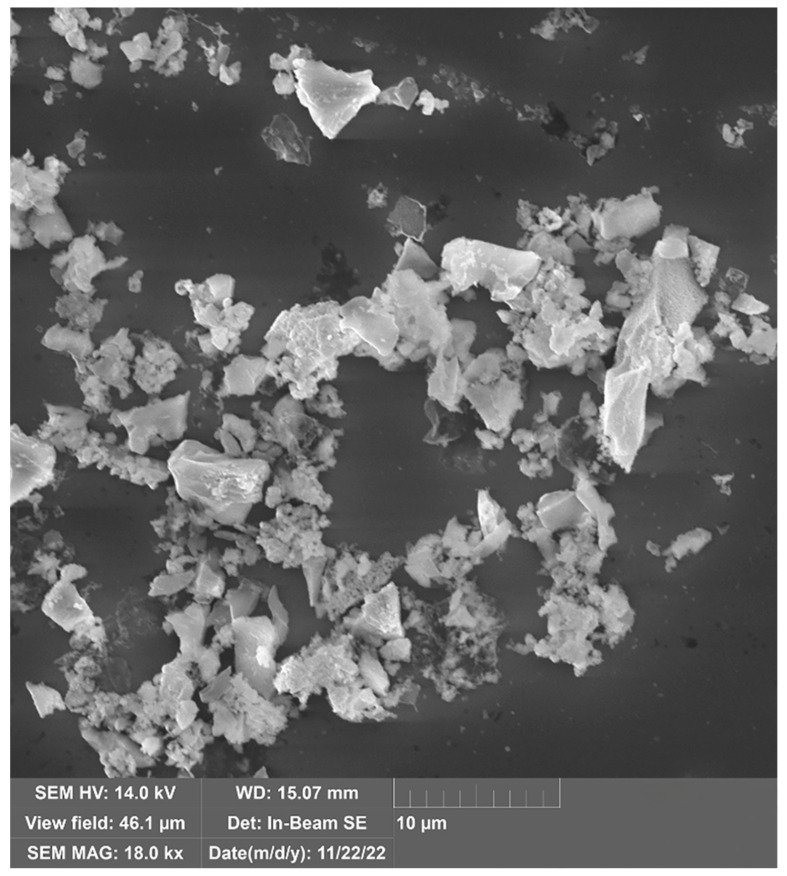
SEM image of SiC particles (5% Al with 6-min reaction time).

Despite its small crystallite size, SEM shows a broad size distribution as particles close the crystallite size and as large as 5 µm can be observed (Figure 2) The EDS analysis of 5% Al samples is summarized in Table 1. According to the EDS analysis, the Si concentration decreases with the increase of the reaction time. The slight decrease of Si can be explained by the previously discussed reaction between SiC and Al. It should be noted, however, that evaporation of Si cannot be either neglected at the reaction temperature. The presence of oxygen can be explained by the relatively large surface area. EDS indicates the success of Al removal as the concentration of Al, other impurities, F and N residue from the Si removal are well below the acceptable detection limit of 1 at%. Nevertheless, Table 1 presents all elements with non-zero concentration from the result of the Monte-Carlo simulation used to calculate the concentrations.

The EPR spectra of the samples show multiple paramagnetic centers. For analysis, the total paramagnetic defect concentration was calculated from the double integrals of the original EPR spectra after sample normalizations with mass and instrumental factors and the EPR spectra were also fitted by using the Hamiltonians of the most abundant defects in SiC. The system can be described with the following Spin-Hamiltonian,

(1) $$H={µ}_{B}SgB+\sum_{i}SA\left(i\right)I\left(i\right)-g_{N\left(i\right)}{µ}_{N\left(i\right)}I\left(i\right)B$$

Here ***g*** is the *g*-tensor (in the most generic case it is a rank two tensor) and *A*$\left(i\right)$ is a hyperfine (HF) tensor for a nuclear spin [29,37]. This equation is amended by the zero-field splitting term for *S >* 1/2 spin systems which reads as SDS. Since our samples are in powder form, a so-called powder-average is used for the *g*-tensor and for the hyperfine tensors. The spin Hamiltonian parameters of the defect centers, V_Si_^−^ [38], V_C_^+^ [29], V_C_-C_Si_^+^ [25] and V_C_-V_Si_ [14] are summarized in Table 2 that are used as an initial set for fitting to the observed EPR spectra. We note that the known EPR parameters in 3C-SiC are very similar to the known parameters in the so-called hexagonal defect configurations in the hexagonal polytypes.

**Figure 3:**
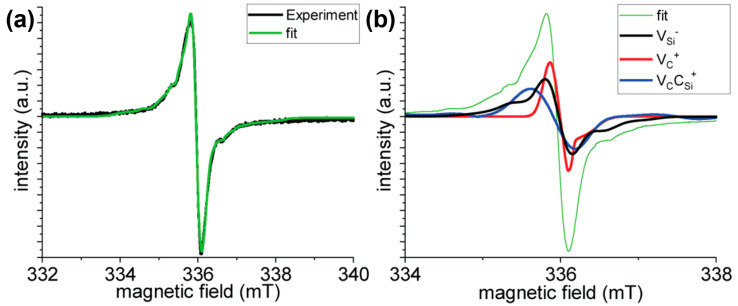
EPR spectrum and its analysis. (**a**) EPR spectrum (black curve) and EPR simulation result (green curve) on the 5%Al sample with 6-min reaction time. (**b**) Contribution of the considered three defect spins to the total EPR spectrum in (**a**).

We found that the features of the neutral divacancy do not show up in the recorded EPR spectrum such as the pair of main peaks caused by the large zero-field-splitting (see Table 2), thus we assumed that its concentration is very low or they do not exist in our samples. As a consequence, we fit the EPR signals of the three basic vacancy defect types to the observed EPR data. With the spin Hamiltonian parameters of the three basic vacancy defects in SiC, the EPR spectra can be simulated relatively well as illustrated for one particular sample in Figure 3a. In the fitting procedure, the effect of hexagonal inclusions in 3C-SiC on the EPR spectra of point defects can be considered as local strains with respect to the perfect 3C-SiC environment. Additional perturbation could be the presence of interface between SiC and the terminating oxide layer that may be also viewed as random strain. These types of strain distort the tetrahedral symmetry of silicon-vacancy and cause a small zero-field-splitting. Furthermore, the main EPR peak of the vacancies (associated with the *g*-factor) and the satellite hyperfine lines are broadened. The broadening can be wide under these circumstances and the hyperfine peaks of few percent with respect to the main EPR peak will be invisible. Therefore, the carbon-related hyperfine peaks of silicon-vacancy may not be recognized in the EPR spectrum. However, the strong Si-related hyperfine peaks (of about 40% with respect to the main EPR peak) should be visible even when broadened. Indeed, the small shoulder in the EPR spectrum originates from the Si-vacancy’s EPR signal which exhibits intense hyperfine satellites from the second neighbor ^29^Si isotopes (c.f. Figure 3a,b). The fit typically results in *D* = 14 MHz constant for the Si-vacancy which is usually characteristic for the EPR signals of Si-vacancies in hexagonal polytypes. The broadened EPR signals of the positively charged carbon antisite-vacancy and carbon-vacancy can also be recognized in the observed EPR spectra (e.g., Figure 3b). The EPR spectrum of carbon-vacancy mostly contributes to the broadening of the main EPR peak, but their hyperfine satellites remain elusive due to the broadening. On the other hand, the hyperfine satellites of the carbon antisite-vacancy pair faintly appear at around 334.5 mT in our EPR setup (see Figure 3b), and this paramagnetic defect also strongly contributes to further broadening of the central EPR peak.

We conclude from this analysis that the presence of silicon-vacancy can be convincingly proven from the EPR spectrum. The features of the positively charged carbon antisite-vacancy pair defect in the EPR spectrum can be also read out. On the other hand, the EPR signal of the carbon-vacancy can only be indirectly implied since it mostly contributes to the broadening of the central EPR peak. We note that the surface dangling bond, the so-called P_bC_ center [39] has a *S* = 1/2 spin state, similar to that of the carbon-vacancy. We found that the P_bC_ center of porous SiC, briefly porous-P_bC_ center in the context [39], cannot account for the broadening of the observed central EPR peak since its *g*-tensor would lead to significantly shift in the EPR spectrum with respect to the observed one. Another type of P_bC_ center has been reported for thermally oxidized 4H-SiC which has been observed by EPR and electrically detected magnetic resonance (EDMR) [40,41]. The line shape of that P_bC_ EPR center is similar to that of our EPR spectra. However, the hyperfine satellites (shoulders in our EPR spectra) are located closer to each other than 1.3 mT observed for the P_bC_ center. Furthermore, we do not use high temperature (1100 °C) thermal oxidation of our SiC particles, and thus it is not likely that we generate the same interface defects that have been observed at the 4H-SiC/SiO_2_ interface. We finally conclude that the most abundant native paramagnetic defect (e.g., see the formation energies of these defects in Ref. [42]), the carbon-vacancy, rather appears in our sample which is manifested as the broadening of the central EPR peak in the EPR spectrum. The association of the shoulder features in the EPR spectrum with the hyperfine satellites of the silicon-vacancy is further corroborated by PL experiments in terms of the existence of silicon-vacancies in our sample that will be presented below.

**Figure 4:**
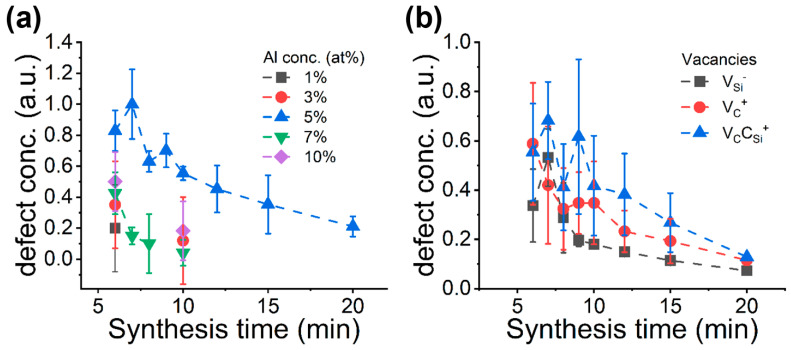
Defect analysis based on the EPR data of paramagnetic defects. (**a**) Integrated defect concentration in different SiC samples and (**b**) the calculated relative defect concentrations from simulation of SiC samples prepared with 5% Al.

We state again that our goal is to maximize the concentration of spin defects the defect qubit spins. In our sample, silicon-vacancies may act as defect qubits. Thus, we investigated its concentration by monitoring and analyzing the EPR spectra. The two basic synthesis parameters are the aluminum (Al) concentration in the precursor and the synthesis time. In each batch, we synthesized at least five samples to study the defect spin concentration which results in a mean value and a standard deviation for each batch. We applied the following strategy in these investigations. First, we identified the optimal aluminum concentration in the precursor as shown in Figure 4a. The result of the relative paramagnetic defect concentrations shows that the maximum defect concentration is reached when 5% Al precursor was added to silicon and carbon sources. We found that the defect spin concentration in the synthesized SiC is always lower at other Al concentrations in the precursor. Figure 4a also shows that the defect concentration is decreasing with increases in reaction time. For other than 5 at% Al concentration the defect spin concentration rapidly decreases. Therefore, we studied the evolution of the defect spin concentration for relatively long reaction times only for 5 at% Al concentration samples. For these samples, the small increase in the defect spin concentration at 7 min indicates defect formation at the early stage of the reaction when nucleation is a dominant process. In the particle growth reaction stage, however, the high reaction temperature anneals out most of the defects created at the early state of reaction. Indeed, XRD data show that at between 5 and 10 min of reaction times, the crystallite size and the non-cubic phase concentration is relatively constant compared to the results from longer reaction times, and with longer than 10 min of reaction time, the dominant reaction is crystal growth and defect annihilation.

Our study confirms that the 5 at% Al is the optimal one to maximize the defect spin concentration in our SiC synthesis. Therefore, we analyze the paramagnetic defect distribution only for these batches as a function reaction time (see Figure 4b). We note here that the carbon antisite-vacancy pair defect is another “face” of silicon-vacancy defect in SiC (i.e., can be achieved by moving one of the nearest carbon atom of the silicon-vacancy into the Si-site [27] where carbon antisite-vacancy pair defect is more stable under intrinsic conditions than silicon-vacancy defect [42]). We further note that carbon-vacancy is more stable when created under thermal equilibrium in SiC than the previously considered vacancies are [37,42]. The simulation shows a high amount of carbon antisite-vacancy pair defect (i.e., the most stable form of Si-deficient defects in SiC) in the samples at the early stage of the reaction and then a rapid decrease with increasing the reaction time. At a smaller amount, the less stable form of Si-deficient defect, silicon-vacancy, also forms, which mostly follows the evolution of the carbon antisite-vacancy pair defect upon reaction times. The highest silicon-vacancy concentration was achieved for 7-min reaction time in our setup which is the most favorable situation for creating defect qubits. Interestingly, the silicon-vacancy concentration is only smoothly declining after longer than 10 min of reaction time. As was already discussed [29], the presence of Al influences the SiC formation in two ways. On the one hand, it promotes hexagonal SiC growth that manifest in the increase of the non-cubic phases due to the hexagonal inclusions or stacking faults. On the other hand, aluminum reacts with SiC at high temperatures. The reaction is selective towards silicon atoms. The relative concentration of the silicon-vacancy shows that despite the continuous annihilation on various reaction routes, the formation rate of silicon-vacancy is high enough to maintain its concentration during SiC growth. Finally, ther is the carbon-vacancy. We first note that the presence of the positively charged carbon-vacancy as *S* = 1/2 spin defect cannot be convincingly proven solely from the analysis of the EPR spectrum. Nevertheless, this defect as the most thermally stable paramagnetic defect likely appears in the synthesis of SiC. Its concentration almost monotonously decreases with longer reaction times as expected.

**Figure 5:**
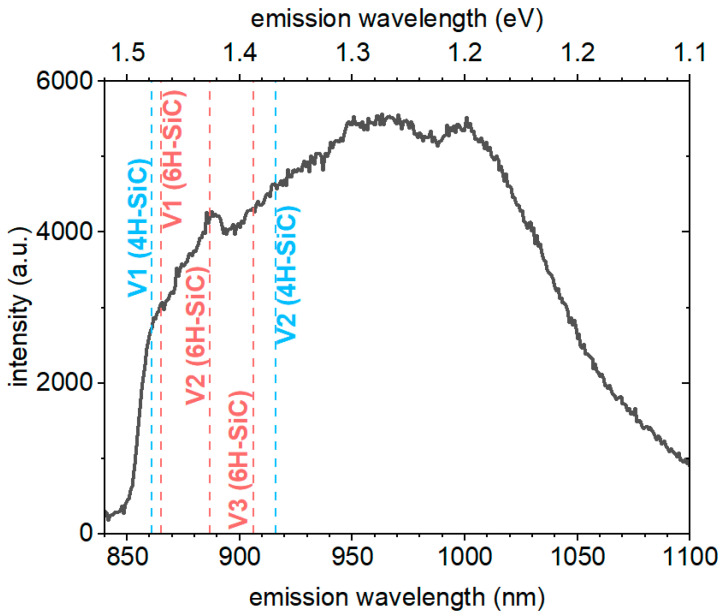
PL spectrum collected from SiC powder made with 5% Al and 15-min reaction time. The zero-phonon-lines of the known V_Si_^−^ centers in different polytypes are marked as vertical lines.

The calculated relative concentrations for the different defects suggest that at 15-min reaction time, the carbon antisite-vacancy defect concentration reaches a minimum, while the silicon-vacancy concentration is sinking at a much lower pace with increasing reaction times and is still well observable at 15-min reaction time. Silicon-vacancy emits in the infrared range of 860–1000 nm. The synthesized samples contain various defects, most of them could contribute to the optical properties of SiC through photon emission or non-radiative relaxation, the high stacking fault concentration and surface moieties due to the 30–50 nm crystallite size could broaden the emission spectra increasing the difficulty of the emission center detection. Nonetheless, we measured the near-infrared emission under 785 nm laser excitation (Figure 5). Despite the detected peaks which positions are coinciding with the zero-phonon-lines and phonon sideband features of V_Si_^−^ in 4H-SiC and 6H-SiC, the overall emission is quite broad at room temperature. Again, the high concentration of hexagonal inclusions in the sample can well explain the observation of the PL emission of silicon-vacancies typical in hexagonal polytypes.

## 5. Conclusions

We successfully synthesized SiC with introduced paramagnetic and fluorescent point defects by adding aluminum precursor in the chemical combustion reaction. We find that 5% Al content to the initial silicon and carbon powders results in the largest concentration of paramagnetic point defects in the synthesized SiC. Monitoring the evolution of paramagnetic point defects in SiC by EPR spectroscopy reveals that the total concentration of paramagnetic point defects shows monotonous decay as a function of reaction time. We find that 6 min reaction time is the optimum one in our setup to maximize the concentration of paramagnetic defects. Among the two established defect quantum bits (the silicon-vacancy and divacancy), the divacancy concentration is always small or divacancies do not form irrespective to the applied synthesis conditions. We suspect that the relatively small temperature and short synthesis times do not permit the diffusion of isolated vacancies to form larger vacancy aggregates such as divacancies. The carbon vacancy EPR signal is observable at all conditions which is the most thermally stable paramagnetic center among the intrinsic defects in SiC. The silicon-vacancy has much higher formation energies than that of carbon-vacancy. Nevertheless, the silicon-vacancy concentration remains relatively high and stable due to the reaction between aluminum and SiC that takes out Si atoms from SiC. In the respective SiC samples, we observe near-infrared fluorescence in the region which is associated with the PL spectra of silicon-vacancies in hexagonal polytypes. The appearance of silicon-vacancies in the PL spectra reaffirms our analysis on the recorded EPR spectra in terms of the origin of the shoulder features. The PL signals associated with the silicon-vacancies in hexagonal polytypes are consistent with the XRD analysis that shows a high density of hexagonal inclusions in cubic SiC that is again promoted by the Al precursor in the chemical reaction.

## Figures and Tables

**Table 1:** EDS analysis result of SiC samples made from precursor containing 5% Al.

	Reaction Time							
	6 min	7 min	8 min	9 min	10 min	12 min	15 min	
Elements	Concentration [at%], (Error%)							
C	39.3 (12.1)	44.0 (11.8)	58.1 (11.5)	54.5 (11.7)	58.6 (11.5)	59.8 (11.4)	60.2 (11.5)	
Si	58.3 (2.6)	51.7 (2.7)	39.2 (2.7)	42.5 (2.9)	36.0 (3.0)	36.4 (2.8)	38.3 (2.7)	
Al	0.2 (8.9)	0.2 (8.8)	0.6 (4.9)	0.2 (6.7)	1.3 (4.5)	0.1 (9.3)	0.1 (9.2)	
O	1.7 (12.2)	3.4 (10.8)	2.1 (11.9)	2.5 (12.3)	3.2 (12.1)	2.6 (12.1)	0.8 (15.9)	
F					0.2 (15.9)			
N				0.4 (43.1)	0.5 (28.9)	0.8 (22.4)	0.6 (34.0)	
Na	0.3 (8.3)					0.1 (12.3)		
K						0.1 (21.5)		
S	0.2 (5.8)							
Fe		0.4 (14.7)			0.1 (29.1)			
Cu		0.3 (25.6)						

**Table 2:** Initial spin Hamiltonian parameters of paramagnetic defects in SiC. We provide the average *g*-factor, the spin state *S* and the hyperfine constants (principal values of the hyperfine tensors). The *D*-constant of divacancy is set to 1300 MHz. For the silicon-vacancy we set it to zero which is valid in 3C-SiC.

Defect	*g*-Factor, *S*	*A*_xx_ (MHz)	*A_yy_* (MHz)	*A_zz_* (MHz)
V_Si_^−^	2.0029, 3/2			
^13^C × 4		33.0	33.0	80.0
^29^Si × 12		8.2	8.2	8.2
V_C_^+^	2.0027, 1/2			
^29^Si × 4		120.0	120.0	180.0
V_C_-C_Si_^+^	2.0035, 1/2			
^13^C × 1		64.0	64.0	231.0
^29^Si × 3		64.0	64.0	64.0
V_C_-V_Si_	2.0030, 1			
^13^C × 3		50.0	50.0	110.0
^29^Si × 3		12.0	12.0	12.0

## Data Availability

Experimental data will be available from Concorda (Concentrated Cooperation on Research Data) repository.

## References

[1] Hemmingsson C., Son N.T., Kordina O., Bergman J.P., Janzén E., Lindström J.L., Savage S., Nordell N. (1997). Deep Level Defects in Electron-Irradiated 4H SiC Epitaxial Layers. J. Appl. Phys..

[2] Chakravorty A., Singh B., Jatav H., Meena R., Kanjilal D., Kabiraj D. (2021). Controlled Generation of Photoemissive Defects in 4H-SiC Using Swift Heavy Ion Irradiation. J. Appl. Phys..

[3] Liu J., Xu Z., Song Y., Wang H., Dong B., Li S., Ren J., Li Q., Rommel M., Gu X. (2020). Confocal Photoluminescence Characterization of Silicon-Vacancy Color Centers in 4H-SiC Fabricated by a Femtosecond Laser. Nanotechnol. Precis. Eng..

[4] Sörman E., Son N.T., Chen W.M., Kordina O., Hallin C., Janzén E. (2000). Silicon Vacancy Related Defect in 4H and 6H SiC. Phys. Rev. B.

[5] Janzén E., Gali A., Carlsson P., Gällström A., Magnusson B., Son N.T. (2009). The Silicon Vacancy in SiC. Phys. B Condens. Matter.

[6] Nagy R., Widmann M., Niethammer M., Dasari D.B.R., Gerhardt I., Soykal Ö.O., Radulaski M., Ohshima T., Vučković J., Son N.T. (2018). Quantum Properties of Dichroic Silicon Vacancies in Silicon Carbide. Phys. Rev. Appl..

[7] Nagy R., Niethammer M., Widmann M., Chen Y.-C., Udvarhelyi P., Bonato C., Hassan J.U., Karhu R., Ivanov I.G., Son N.T. (2019). High-Fidelity Spin and Optical Control of Single Silicon-Vacancy Centres in Silicon Carbide. Nat. Commun..

[8] Ivády V., Davidsson J., Son N.T., Ohshima T., Abrikosov I.A., Gali Á. (2018). *Ab Initio* Theory of Si-Vacancy Quantum Bits in 4H and 6H-SiC. Mater. Sci. Forum.

[9] Kraus H., Soltamov V.A., Riedel D., Väth S., Fuchs F., Sperlich A., Baranov P.G., Dyakonov V., Astakhov G.V. (2013). Room-Temperature Quantum Microwave Emitters Based on Spin Defects in Silicon Carbide. Nat. Phys..

[10] Widmann M., Lee S.-Y., Rendler T., Son N.T., Fedder H., Paik S., Yang L.-P., Zhao N., Yang S., Booker I. (2014). Coherent Control of Single Spins in Silicon Carbide at Room Temperature. Nat. Mater..

[11] Echlin P. (2009). Handbook of Sample Preparation for Scanning Electron Microscopy and X-ray Microanalysis.

[12] Niethammer M., Widmann M., Rendler T., Morioka N., Chen Y.-C., Stöhr R., Hassan J.U., Onoda S., Ohshima T., Lee S.-Y. (2019). Coherent Electrical Readout of Defect Spins in Silicon Carbide by Photo-Ionization at Ambient Conditions. Nat. Commun..

[13] Carlos W.E., Garces N.Y., Glaser E.R., Fanton M.A. (2006). Annealing of Multivacancy Defects in 4H−SiC. Phys. Rev. B.

[14] Son N.T., Carlsson P., ul Hassan J., Janzén E., Umeda T., Isoya J., Gali A., Bockstedte M., Morishita N., Ohshima T. (2006). Divacancy in 4H-SiC. Phys. Rev. Lett..

[15] Gali A., Gällström A., Son N.T., Janzén E. (2010). Theory of Neutral Divacancy in SiC: A Defect for Spintronics. Mater. Sci. Forum.

[16] Falk A.L., Buckley B.B., Calusine G., Koehl W.F., Dobrovitski V.V., Politi A., Zorman C.A., Feng P.X.-L., Awschalom D.D. (2013). Polytype Control of Spin Qubits in Silicon Carbide. Nat. Commun..

[17] Ivády V., Szász K., Falk A.L., Klimov P.V., Janzén E., Abrikosov I.A., Awschalom D.D., Gali Á. (2016). First Principles Identification of Divacancy Related Photoluminescence Lines in 4H and 6H-SiC. Mater. Sci. Forum.

[18] Koehl W.F., Buckley B.B., Heremans F.J., Calusine G., Awschalom D.D. (2011). Room Temperature Coherent Control of Defect Spin Qubits in Silicon Carbide. Nature.

[19] Christle D.J., Falk A.L., Andrich P., Klimov P.V., Hassan J.U., Son N.T., Janzén E., Ohshima T., Awschalom D.D. (2014). Isolated Electron Spins in Silicon Carbide with Millisecond Coherence Times. Nat. Mater..

[20] Magnusson B., Janzén E. (2005). Optical Characterization of Deep Level Defects in SiC. Mater. Sci. Forum.

[21] Somogyi B., Gali A. (2014). Computational Design of *in Vivo* Biomarkers. J. Phys. Condens. Matter.

[22] de Vries M.O., Sato S., Ohshima T., Gibson B.C., Bluet J., Castelletto S., Johnson B.C., Reineck P. (2021). Fluorescent Silicon Carbide Nanoparticles. Adv. Opt. Mater..

[23] Castelletto S., Johnson B.C., Ivády V., Stavrias N., Umeda T., Gali A., Ohshima T. (2013). A Silicon Carbide Room-Temperature Single-Photon Source. Nat. Mater..

[24] Castelletto S., Johnson B.C., Zachreson C., Beke D., Balogh I., Ohshima T., Aharonovich I., Gali A. (2014). Room Temperature Quantum Emission from Cubic Silicon Carbide Nanoparticles. ACS Nano.

[25] Umeda T., Ishoya J., Ohshima T., Morishita N., Itoh H., Gali A. (2007). Identification of Positively Charged Carbon Antisite-Vacancy Pairs in 4H-SiC. Phys. Rev. B.

[26] von Bardeleben H.J., Rauls E., Gerstmann U. (2020). Carbon Vacancy-Related Centers in 3C-Silicon Carbide: Negative-U Properties and Structural Transformation. Phys. Rev. B.

[27] Rauls E., Lingner T., Hajnal Z., Greulich-Weber S., Frauenheim T., Spaeth J.-M. (2000). Metastability of the Neutral Silicon Vacancy in 4H-SiC. Phys. Status Solidi B.

[28] Deák P., Aradi B., Frauenheim T., Gali A. (2008). Challenges for Ab Initio Defect Modeling. Mater. Sci. Eng. B.

[29] Umeda T., Isoya J., Morishita N., Ohshima T., Kamiya T., Gali A., Deák P., Son N.T., Janzén E. (2004). EPR and Theoretical Studies of Positively Charged Carbon Vacancy in 4H-SiC. Phys. Rev. B.

[30] Beke D., Valenta J., Károlyházy G., Lenk S., Czigány Z., Márkus B.G., Kamarás K., Simon F., Gali A. (2020). Room-Temperature Defect Qubits in Ultrasmall Nanocrystals. J. Phys. Chem. Lett..

[31] Beke D., Szekrényes Z., Czigány Z., Kamarás K., Gali Á. (2015). Dominant Luminescence Is Not Due to Quantum Confinement in Molecular-Sized Silicon Carbide Nanocrystals. Nanoscale.

[32] Viala J.C., Bosselet F., Laurent V., Lepetitcorps Y. (1993). Mechanism and Kinetics of the Chemical Interaction between Liquid Aluminium and Silicon-Carbide Single Crystals. J. Mater. Sci..

[33] Du H., Yang Z., Libera M., Jacobson D.C., Wang Y.C., Davis R.F. (1993). Chemistry and Structure of Beta Silicon Carbide Implanted with High-Dose Aluminum. J. Am. Ceram. Soc..

[34] Beke D., Károlyházy G., Czigány Z., Bortel G., Kamarás K., Gali A. (2017). Harnessing No-Photon Exciton Generation Chemistry to Engineer Semiconductor Nanostructures. Sci. Rep..

[35] Mukasyan A.S., Lin Y.-C., Rogachev A.S., Moskovskikh D.O. (2013). Direct Combustion Synthesis of Silicon Carbide Nanopowder from the Elements. J. Am. Ceram. Soc..

[36] Stoll S., Schweiger A. (2006). EasySpin, a Comprehensive Software Package for Spectral Simulation and Analysis in EPR. J. Magn. Reson..

[37] Bockstedte M., Heid M., Pankratov O. (2003). Signature of Intrinsic Defects in SiC: Ab Initio Calculations of Hyperfine Tensors. Phys. Rev. B.

[38] Umeda T., Morishita N., Ohshima T., Itoh H., Isoya J. (2007). Electron Paramagnetic Resonance Study of Carbon Antisite-Vacancy Pair in p-Type 4H-SiC. Mater. Sci. Forum.

[39] Cantin J.L., Von Bardeleben H.J., Shishkin Y., Ke Y., Devaty R.P., Choyke W.J. (2004). Identification of the Carbon Dangling Bond Center at the 4H−SiC/SiO2 Interface by an EPR Study in Oxidized Porous SiC. Phys. Rev. Lett..

[40] Umeda T., Kobayashi T., Sometani M., Yano H., Matsushita Y., Harada S. (2020). Carbon Dangling-Bond Center (Carbon *P* b Center) at 4H-SiC(0001)/SiO_2_ Interface. Appl. Phys. Lett..

[41] Umeda T., Nakano Y., Higa E., Okuda T., Kimoto T., Hosoi T., Watanabe H., Sometani M., Harada S. (2020). Electron-Spin-Resonance and Electrically Detected-Magnetic-Resonance Characterization on *P* _bC_ Center in Various 4H-SiC(0001)/SiO_2_ Interfaces. J. Appl. Phys..

[42] Szász K., Ivády V., Abrikosov I.A., Janzén E., Bockstedte M., Gali A. (2015). Spin and Photophysics of Carbon-Antisite Vacancy Defect in 4 H Silicon Carbide: A Potential Quantum Bit. Phys. Rev. B.

